# A review of sleep-related indicators measured in large population-based cohort studies in Japan

**DOI:** 10.1265/ehpm.25-00277

**Published:** 2026-04-03

**Authors:** Kenichi Ariyada, Kazumasa Yamagishi, Jaehoon Seol, Masashi Yanagisawa, Masao Iwagami

**Affiliations:** 1 International Institute for Integrative Sleep Medicine, University of Tsukuba, Tsukuba, Japan; 2 Department of Digital Health, Institute of Medicine, University of Tsukuba, Tsukuba, Japan; 3 Health Services Research and Development Center, University of Tsukuba, Tsukuba, Japan; 4 Department of Public Health Medicine, Institute of Medicine, University of Tsukuba, Tsukuba, Japan; 5 Department of Public Health, Graduate School of Medicine, Juntendo University, Tokyo, Japan; 6 Institute of Health and Sport Sciences, University of Tsukuba, Tsukuba, Japan; 7 Department of Molecular Genetics, University of Texas Southwestern Medical Center, Dallas, Texas, USA

**Keywords:** Sleep, Sleep measures, Cohort study, Actigraphy, Pulse oximetry

## Abstract

**Background:**

How to measure sleep has been the primary focus of sleep epidemiological studies. To address the lack of systematic evaluation of sleep-related indicators, we reviewed large population-based cohort studies in Japan, focusing on whether sleep was measured subjectively or objectively.

**Methods:**

PubMed, Ichushi-Web, and official cohort study websites were systematically searched from January 1, 2004, to March 31, 2025, to identify studies that measured sleep-related indicators as exposures or outcomes in 19 large population-based cohorts in Japan. Data on sleep measurement methods and main study results were extracted.

**Results:**

Of 102 studies in 12 cohorts, 75 (74%) studies examined subjective sleep quantity or quality based on questionnaires, and 27 (26%) used objective measures of sleep. Among subjective measures, sleep duration was frequently self-reported (57 studies in 10 cohorts), while sleep quality was directly (e.g., “Do you think you get enough sleep?”) or indirectly asked as part of sleep-related scores such as the Pittsburgh Sleep Quality Index (51 studies in 10 cohorts). Regarding objective measures, the Circulatory Risk in Communities Study cohort measured 3% oxygen desaturation index by pulse oximetry (9 studies) and respiratory disturbance index by airflow monitor (1 study), Japan Multi-Institutional Collaborative Cohort Study used actigraphy (1 study), Nagahama cohort used pulse oximetry (12 studies) and actigraphy (13 studies), and Tohoku Medical Megabank Project used actigraphy (1 study) and contactless biomotion sleep sensor (2 studies).

**Conclusions:**

Population-based cohort studies in Japan have predominantly relied on subjective measures of sleep, while the number of studies using objective measures has been increasing.

**Supplementary information:**

The online version contains supplementary material available at https://doi.org/10.1265/ehpm.25-00277.

## Background

Sleep duration and quality and sleep-wake patterns are risk factors for all-cause mortality and cardiometabolic disorders in the general population [1–8]. Sleep deficiency impairs work performance and driving [9–11]. Generating high-quality evidence by prospectively evaluating the association between sleep and health outcomes is crucial for assessing the health and social burden of sleep disorders.

Most epidemiological studies have collected subjective data on sleep duration and quality using self-reported questionnaires to evaluate the association of these variables with the incidence of various diseases and mortality. However, several epidemiological studies in the United States and European countries have assessed sleep using objective tools such as actigraphy [1, 12–16]. A systematic review conducted by the American Academy of Sleep Medicine demonstrated that actigraphy was a useful and low-cost objective measure of sleep patterns and parameters across a wide range of sleep disorders [17].

Japanese people are known for having short sleep duration, particularly women, in contrast to the patterns observed in other countries [18]. However, the tools and methods used to assess sleep have not been systematically reviewed, although there have been a number of large population-based cohort studies in Japan.

Therefore, this study (1) reviewed large population-based cohort studies that subjectively or objectively assessed sleep in the Japanese general population, (2) identified what sleep-related indicators were measured, and (3) summarizes the main findings on the association of sleep measures and the outcome or exposure of interest.

## Methods

### Review of large population-based cohort studies in Japan

We first defined and identified large population-based cohorts in Japan, and then systematically reviewed studies conducted within the cohorts. In reference to the definitions in the Japan Epidemiological Association registry [19], we identified population-based cohorts wherein (i) the number of participants at the baseline survey exceeded 10,000, and (ii) the follow-up period was at least 5 years. Consequently, we identified 19 cohorts: Japan Public Health Center (JPHC) Study (Cohorts I and II); The Japan Collaborative Cohort (JACC) Study; The Miyagi Cohort Study; Ohsaki National Health Insurance Cohort Study; The Three Prefecture Study (Aichi, Miyagi, and Osaka); Takayama Study; Life Span Study; Circulatory Risk in Communities Study (CIRCS); NIPPON DATA; Ibaraki Prefectural Health Study; Komo-Ise Gunma Cohort Study; Japan Multi-Institutional Collaborative Cohort (J-MICC) Study; Japan Nurses’ Health Study (JNHS); The Hokkaido Study on Environment and Children’s Health; and the Japanese epidemiological study on low-dose radiation effects (J-EPISODE); the JPHC for the Next Generation (JPHC-NEXT) Study; the Japan Environment and Children’s Study (JECS); the Nagahama Study; and the Tohoku Medical Megabank Project (TMM) Cohort Study.

### Literature review

Based on these 19 cohorts, we systematically searched PubMed, Ichushi-Web (bibliographic database of Japanese biomedical information), and the official websites for studies published between January 2004 and March 2025. We used the search terms “sleep OR sleep [MeSH]” in combination with the name of the cohort study in PubMed and performed a similar search in Ichushi-Web. Exceptionally, the CIRCS was first named in 2008, and therefore, studies published before 2008 were searched by names of the authors in the CIRCS studies published after 2008. In addition, we reviewed the publication lists on official websites to identify studies that may have been missed during our PubMed and Ichushi-Web searches.

The inclusion criteria were studies that collected and used sleep data, regardless of the study design. The exclusion criteria were (i) studies wherein sleep-related indicators were neither an exposure nor outcome of interest (e.g., a sleep-related indicator was considered a confounder or included as a component of a lifestyle-related risk score) and (ii) review studies. Eligible studies were screened by title, abstract, and full text. The first author (KA) selected eligible studies, and the last author (MI) checked the selection process. Disagreements were resolved by consensus.

### Extracted information

Among the selected studies, we extracted the following information: name of the cohort, title of the study, name of the first author, publication year, number of participants, study design, study outcomes, sleep measures, and main findings of the study.

### Data analysis and presentation

We provided details of the selected studies. We counted the number of studies in each cohort, the distribution of study designs, and the methods used to measure sleep. We conducted a chi-square trend test on whether the proportion of cohort studies using objective sleep measures (among all cohort studies using either subjective or objective sleep measures) changed over time, wherein years were grouped as 2010 or before, 2011–2015, 2016–2020, and 2021–2025. P-value of <0.05 was regarded as statistically significant. Ethical approval was waived because the study was based on publicly available information.

## Results

### Selection of eligible studies

**Fig. 1:**
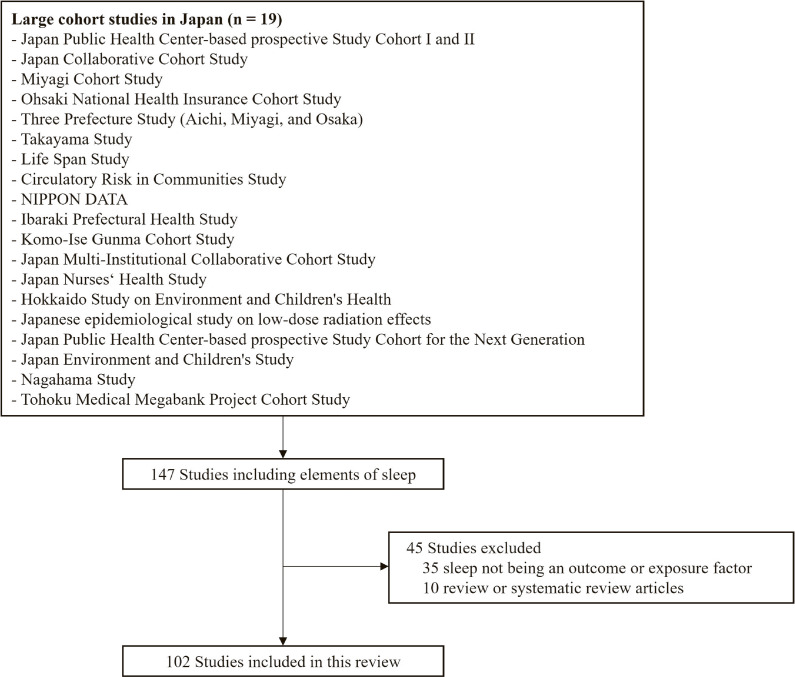
Flowchart of study selection.

Details on the study selection process are shown in Fig. 1. A systematic search identified 147 studies that met the inclusion criteria. Then, we excluded 35 studies wherein sleep parameters were neither an exposure nor an outcome of interest, as well as 10 literature reviews. A total of 102 studies in 12 cohorts were included in the review, as follows: 3 studies in the JPHC Study Cohorts I and II [20–22], 12 studies in the JACC Study [23–34], 5 studies in the Ohsaki National Health Insurance Cohort Study [35–39], 3 studies in the Takayama Study [40–42], 12 studies in the CIRCS [43–54], 10 studies in the J-MICC Study [55–64], 3 studies in the JNHS [65–67], 2 studies in the J-EPISODE [68, 69], 2 studies in the JPHC-NEXT [70, 71], 24 studies in the JECS [72–95], 19 studies in the Nagahama Study [96–114], and 7 studies in the TMM Cohort Study [115–121].

The studies are summarized in Table S1, and the distributions of the cohorts and sleep parameters are shown in Table 1. Of 102 studies, 51 (50%) had a cross-sectional design, and 51 (50%) had a longitudinal design; 75 (74%) studies examined subjective sleep quantity or quality based on questionnaires, and 27 (26%) used objective measures of sleep; and 73 (72%) and 29 (28%) studies regarded sleep parameters as exposures- and outcomes of interest, respectively.

**Table 1:** Summary of large cohort studies on sleep in Japanese populations.

**Cohorts**	**Number of publications**	**Sleep-related parameters**		**If the sleep parameter was the exposure ** **of interest, what was the study outcome(s)**	**If the sleep parameter was the outcome ** **of interest, what was the study exposure**
		**Subjective**	**Objective**		
Japan Public Health Center-based prospective Study Cohort I and II	3	Sleep duration (ref 20–22) Changes in sleep duration (ref 22)	None	All-cause mortality and major cause-specific mortality (ref 21) Epithelial ovarian cancer (ref 20) Dementia requiring care (ref 22)	None
Japan Collaborative Cohort Study	12	Sleep duration (ref 23–34) Napping (ref 27, 30–32, 34)	None	All-cause mortality (ref 23, 25) Mortality from cardiovascular disease and other causes (ref 24, 27) Mortality from aortic disease (ref 34) Mortality from chronic kidney disease (ref 33) Life expectancy (ref 26, 29) Breast cancer (ref 28) Liver cancer (ref 30) Gastric cancer (ref 32) Type 2 diabetes (ref 31)	None
Ohsaki National Health Insurance Cohort Study	5	Sleep duration (ref 35–39)	None	Cause-specific mortality (ref 38) Prostate cancer (ref 35) Breast cancer (ref 36) Endometrial cancer (ref 39) Weight gain (ref 37)	None
Takayama Study	3	Sleep duration (ref 41, 42) PSQI (ref 40)	None	Mortality from stroke (ref 41) Onset of menopause (ref 42)	Tinnitus (ref 40)
Circulatory Risk in Communities Study	12	Snoring (ref 46, 51, 53) Apnea (ref 46) Excessive daytime sleepiness (ref 46) Morning sleepiness (ref 46)	3% ODI by pulse oximetry (ref 43–49, 52, 54) Respiratory disturbance index by airflow monitor (ref 50)	Cardiovascular disease (ref 53, 54) Blood pressure (ref 43, 46) Atrial fibrillation (ref 45) Type 2 diabetes (ref 47) Metabolic syndrome (ref 48) C reactive protein (ref 49) Excessive daytime sleepiness (ref 46)	Comparison of the sleep-disordered breathing prevalence in Japan and the United States (ref 50) Alcohol consumption (ref 44, 52) Lifestyle Factors (ref 51)
Japan Multi-Institutional Collaborative Cohort Study	10	Sleep duration (ref 55–59, 62, 64) Sleep regularity (ref 55, 58, 59, 62) Sleep quality (ref 62) Bedtime (ref 58) Hypnotics use (ref 60) PSQI (ref 56, 61–64) ISI (ref 64) MEQ (ref 64)	Sleep duration by actigraphy (ref 64) Sleep efficiency by actigraphy (ref 64)	All-cause mortality (ref 59, 60) Sympathetic nervous system activity and bone mass (ref 56) Metabolic syndrome (ref 57, 58) Falls (ref 61) Locomotive syndrome (ref 63)	Dysphagia (ref 62) Forest walks (ref 64) Dietary factors (ref 55)
Japan Nurses’ Health Study	3	Sleep duration (ref 65) Insomnia (ref 66) ESS (ref 67)	None	Lifestyle factors (ref 65) Poor memory or forgetfulness (ref 66)	Shift work (ref 67)
Japanese epidemiological study on low-dose radiation effects	2	Sleep quality (ref 68, 69)	None	Mortality from cancer (ref 68) Mortality from non-cancer (ref 69)	None
Japan Public Health Center-based prospective Study for the Next Generation	2	Sleep duration (ref 70, 71) Sleep quality (ref 70, 71)	None	Dry eye disease (ref 70, 71)	None
Japan Environment and Children's Health	24	**Mothers** Sleep duration (ref 73, 74, 76, 77, 79–81, 85) Wake-up time (ref 74) Bedtime (ref 74, 77, 80, 81, 85) Wake-up mood (ref 73, 77, 80, 81) Sleep quality (ref 74) Sleep depth (ref 74, 77, 80, 81) **Children** Sleep duration (ref 75, 78, 82–84, 86, 88, 89, 92–94) Wake-up time (ref 91) Bedtime (ref 78, 83, 91) Sleeping place and posture (ref 72, 95) Awakenings during the night (ref 77, 78, 80, 81, 83, 87, 90, 93) Crying during the night (ref 78, 83) Sleeping longer during the day than at night (ref 77, 80, 81, 87, 90, 93)	None	**Mothers** Gestational diabetes (ref 76) **Children** Allergic diseases (ref 84) Small for gestational age infants (ref 73, 79) Preterm birth (ref 77) Low birth weight (ref 79) Macrosomia (ref 79) Sleep (ref 77, 80, 81) Temperament (ref 77, 87) Developmental problems (ref 80, 81) Autism spectrum disorder (ref 85, 90, 93) Height (ref 94) Bruxism (ref 89) Otitis media (ref 95)	**Mothers** Fermented food consumption (ref 75, 86) Fish consumption (ref 82) Comparison of sleep status during preconception and pregnancy (ref 74) Physical activity (ref 81) Maternal hemoglobin levels (ref 83) **Children** Non-reassuring fetal status (ref 78) Yogurt and cheese consumption (ref 88) Screen viewing time (ref 84) Neonatal phototherapy (ref 91) Isolated orofacial clefts (ref 92)
Nagahama Study	19	Seep duration (ref 96–98, 100, 102) Sleeplessness (ref 96) Sleepiness (ref 96, 98) Sleep quality (ref 97, 98, 100, 107, 110) Sleep regularity (ref 97, 98) Snoring (ref 104) Stop breathing (ref 104) Hypnotics use (ref 98, 100, 110) ESS (ref 99, 103–105, 107) PSQI (ref 102, 103, 107, 109) Diagnosed sleep apnea syndrome (ref 102, 114)	Seep durations by actigraphy (ref 99, 101, 103–109, 111–114) 3% ODI by pulse oximetry (ref 99, 103–109, 111–114) Sleep efficiency by actigraphy (ref 101, 108, 111) Fragmentation index by actigraphy (ref 101)	Diabetes (ref 99, 105) Blood pressure (ref 99, 101, 111) Lower urinary tract symptoms (ref 100) Nocturnal urination frequency (ref 102) Cardiovascular disease (ref 106) Urinary albumin excretion (ref 108) Serum uric acid (ref 113) Nocturia (ref 110) Metabolome (ref 112) Hemoglobin A1c levels (ref 109) Asthma (ref 114)	Plasma BNP (ref 96) Knee pain and low back pain (ref 97) Combined association of clinical and lifestyle factors (ref 98) Metabolic comorbidities (ref 103) Night-time frequency of urination (ref 104) Differences between subjective and objective sleep duration (ref 107)
Tohoku Medical Megabank Project Cohort Study	7	Seep duration (ref 117) Sleepiness (ref 117) AIS (ref 118, 119, 121)	Sleep efficiency by contactless biomotion sleep sensor (ref 115, 116) Sleep–wake conditions by non-clinical actigraphy device (ref 120)	Hypertension (ref 115, 116) Depression (ref 118)	Social isolation (ref 119) Multiple genetic variants in orexin receptor-2 (ref 117) Heart rate variability (ref 120) Low hemoglobin levels and elevated inflammatory hematological ratios (ref 121)

The reference numbers in the table correspond to those in Table S1.

Abbreviation: PSQI, Pittsburgh sleep quality index; ODI, oxygen desaturation index; ISI, insomnia severity index; MEQ, morningness-eveningness questionnaire; ESS, Epworth sleepiness scale; BNP, B-type natriuretic peptide; AIS, Athens Insomnia Scale.

**Fig. 2:**
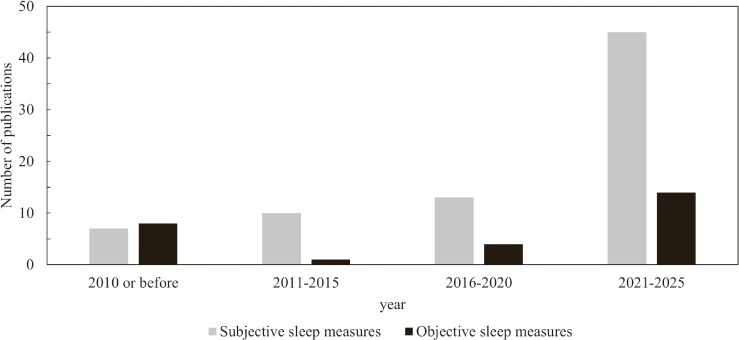
Number of cohort studies by 5-year period and by type of measurements. The figure shows the number of cohort studies on sleep published from January 1, 2004, to March 31, 2025, categorized by 5-year period and by type of measurements (subjective or objective). Studies published in or before 2010 were grouped into a single category.

The temporal trend in the number of cohort studies that measured sleep, subjectively or objectively, is shown in Fig. 2. Several studies from the CIRCS investigating sleep-disordered breathing (as an objective measure) were published before 2010, but later studies predominantly relied on subjective measures. However, the number of studies using objective measures of sleep has increased in recent years. The proportion of studies using objective sleep measures (among all cohort studies using either subjective or objective sleep measures) was 53% (8/15) in 2010 or before, 9% (1/11) in 2011–2015, 24% (4/17) in 2016–2020, and 24% (14/59) in 2021–2025, showing a p-for-trend of 0.118. As a post hoc analysis excluding 2010 or before, a p-for-trend was 0.356.

### Details of subjective sleep measures

Of the 75 studies that used subjective measures, 57 evaluated sleep duration by asking the following questions: “How many hours do you usually sleep per day?” [22] and “How many hours on average did you sleep per day during the last year?” [35]. Sleep duration was either self-reported or calculated based on self-reported bedtime and wake-up times. Researchers often categorized the sleep duration into several groups for statistical analysis; however, the cut-offs for each category differed across studies, and some studies set the reference value to approximately 7 h (Table S1) [122–124].

Of the 75 studies, 51 evaluated subjective sleep quality by asking the questions “Do you think you get enough sleep?” [62] and “Do you get adequate rest during sleep?” [107], as well as by asking questions on the use of hypnotics [60, 98, 100, 110], and symptoms of insomnia [66]. Napping was considered a sleep-related factor [27, 30–32, 34]. Snoring is an indicator of sleep-disordered breathing [46, 51, 53, 104]. Some studies used score-based evaluations, including the Pittsburgh Sleep Quality Index (PSQI), the Insomnia Severity Index (ISI), the Morningness-Eveningness Questionnaire (MEQ), the Epworth Sleepiness Scale (ESS), and the Athens Insomnia Scale (AIS). The PSQI was used as an indicator of sleep quality in the J-MICC Study, Takayama Study, and Nagahama Study [40, 56, 61–64, 102, 103, 107, 109]. The ISI and MEQ, which assess insomnia severity and chronotype, respectively, were used in the J-MICC Study [64]. The ESS was adopted as a scale for measuring daytime sleepiness severity in the JNHS and Nagahama Study [67, 99, 103–105, 107, 109]. The AIS was used as a diagnostic tool for insomnia in the TMM Cohort Study [118, 119, 121].

### Details of objective sleep measures

Twenty-seven studies used objective measures in four cohorts, including the CIRCS, J-MICC Study, Nagahama Study, and TMM Cohort Study. In the CIRCS and Nagahama Study, the 3% oxygen desaturation index (3% ODI), that is the average number of oxygen desaturation measurements of 3% or more per hour during sleep [44, 125], was measured using pulse oximetry in a large cohort of approximately 1,500–4,000 and 7,000 participants, respectively [46–49, 52, 54, 99, 103–109, 111–114]. A study from the CIRCS compared the prevalence of sleep-disordered breathing in Japan and the United States using the respiratory disturbance index measured by airflow monitoring [50].

Actigraphy was used to measure sleep duration in 1 study from the J-MICC Study (approximately 2,000 participants) and 13 studies from the Nagahama Study (approximately 7,000 participants) [64, 99, 101, 103–109, 111–114]. One study from the J-MICC Study and three studies from the Nagahama Study measured sleep efficiency using actigraphy [64, 101, 108, 111]. Participants were instructed to wear the device 24 h a day for 1 week. Daily sleep duration and sleep efficiency were calculated, and bedtime and wake-up times were determined from sleep diaries. Sleep efficiency was defined as the ratio of actual to total sleep duration. Two studies from the TMM Cohort Study used a contactless biomotion sleep sensor as an objective measure to assess sleep efficiency [115, 116].

### Summary of findings on the association between sleep-related measures and outcomes

Among 73 studies that considered sleep-related indicators as exposures of interest, mortality was the most frequent outcome of interest. Most of these studies showed that approximately 7 h of sleep were negatively associated with mortality, regardless of the cause of death [21, 23–25, 33, 38, 41, 59]. One study reported that sleeping 6 h or less was associated with a reduced risk of hemorrhagic stroke mortality in men [41]. Two studies suggested that sleep duration around 7 h was associated with longer life expectancy [26, 29]. Napping was also associated with cardiovascular and non-cardiovascular mortality [27, 34].

Cancer was a common outcome of interest. Sufficient sleep was negatively associated with ovarian, breast, and prostate cancer but not associated with endometrial cancer [20, 28, 35, 36, 39]. The melatonin pathway may underlie the association between sleep duration and cancers [126–128]. Napping was also associated with liver and gastric cancer, although the underlying mechanism was considered unclear [30, 32]. Nocturnal intermittent hypoxia and snoring were associated with hypertension, type 2 diabetes, other metabolic risk factors, and cardiovascular diseases [43, 46, 48, 49, 53, 54, 99, 105, 106]. Sympathetic nervous system activation and intrathoracic negative pressure are potential underlying mechanisms.

The JECS discussed the impact of sleep on maternal and child health as well as on child growth and development. The outcomes included low birth weight in infants, autism spectrum disorder in children, and child height [79, 85, 90, 94]. The findings emphasized the importance of adequate sleep for mothers and children.

### Summary of findings on the association between exposures and sleep

Twenty-nine studies that considered sleep parameters as outcomes of interest assessed exposure factors potentially associated with sleep disturbances. Tinnitus [40], alcohol consumption [44, 52], shift work [67], knee and low back pain [97], and nocturia frequency [104] were associated with sleep problems.

## Discussion

This study reviewed studies that assessed sleep in 19 large population-based cohorts in Japan. Of 102 studies on sleep in 12 cohorts, 75 (74%) used subjective measures based on questionnaires about sleep duration and quality, while 27 (26%) studies used objective measures, such as sleep-disordered breathing by pulse oximetry and sleep duration via actigraphy. The number of studies that use objective measures has been increasing after 2010, although the proportion of them (among all the studied cohort studies) has not increased significantly, mainly because the number of studies that use subjective measures has been also increasing. In the context of growing interest in sleep assessment tools for public health [129], this is the first study to systematically review sleep-related indicators in large population-based cohorts, focusing on the use of subjective and objective measures.

Many cohort studies have examined the association between sleep duration and mortality or the incidence of non-communicable diseases. While many studies in this review collected sleep data using questionnaires, the J-MICC and Nagahama Study evaluated sleep using actigraphy [64, 99, 101, 103–109, 111–114]. Two studies have shown that actigraphy can objectively measure sleep and is generally more accurate than self-reported sleep duration [17, 130]. Another study reported that subjective sleep duration was longer than objective sleep duration when the latter was less than 7 h and shorter when it was 7 h or more [107]. Objective measurements of sleep duration should be encouraged because they are less prone to misclassification.

Sleep quality has been evaluated using several questionnaires. A few cohort studies have adopted score-based evaluations. For instance, the PSQI score was used as an exposure indicator due to its high validity in sleep assessment, and a Japanese version of this index is available [131, 132]. Except for studies that evaluated sleep efficiency (one aspect of sleep quality) using actigraphy [64, 101, 108, 111], other studies did not objectively evaluate sleep quality, which can be accurately measured using a portable electroencephalogram. A retrospective study has shown that over 50% of individuals with sleep disturbances show no abnormalities in electroencephalography-based sleep quality, while nearly 50% of individuals who feel well-rested may have insufficient sleep [133]. These findings underscore the importance of objectively assessing sleep quality.

Sleep-disordered breathing can significantly impact sleep quality. The CIRCS and Nagahama Study evaluated snoring subjectively using questionnaires or objectively using the 3% ODI (measured by pulse oximetry) or respiratory disturbance index (measured by airflow monitoring). Polysomnography is considered the gold standard for diagnosing sleep-related breathing disorders [134], and pulse oximetry alone is insufficient for its definitive diagnosis [135]. However, tests used in community settings should be simpler, faster, and cheaper than clinical tests. Consequently, there is a need to develop simple devices capable of measuring multiple parameters in the general population.

Several epidemiological studies have measured sleep stages using polysomnography, which is considered the gold standard [13, 136, 137]. Accelerometer-based methods that measure 24-h activity and regularity (e.g., sleep regularity index) have become increasingly common, and several longitudinal studies have measured 24-h activity in large cohorts such as those from the UK Biobank and the US National Health and Nutrition Examination Survey. Nonetheless, few longitudinal studies have evaluated these parameters in Japan.

The psychomotor vigilance task, which measures behavioral alertness, is an objective indicator of sleepiness and sleep debt resulting from sleep-disordered breathing. The results of the community-based Toon Study involving approximately 2,500 participants in Japan found that intermittent hypoxia caused by sleep-disordered breathing was significantly associated with poorer performance in the psychomotor vigilance test [138]. With their simple design and online accessibility, the psychomotor vigilance test is expected to be increasingly used in large cohort studies. In addition, the widespread availability of portable electroencephalography devices has enabled the collection of large amounts of data on sleep in Japan [139]. Unlike conventional polysomnography, which typically requires participants to sleep in unfamiliar environments such as hospitals or laboratories with multiple electrodes attached to the body and measures clinical parameters during one to three consecutive nights [140], portable electroencephalography devices enable continuous measurements in the comfort of one’s own home [139]. Therefore, the use of these devices is expected to increase.

Data on sleep are crucial for preventing sleep-related adverse events and the resulting societal losses. Data on sleep duration, sleep quality, and sleep-disordered breathing are important regardless of geographical region and study design. Large cohort studies in Japan have contributed to this body of evidence. However, many studies have evaluated sleep data subjectively in Japan and other countries [6]. Subjective sleep assessments may be insufficient for measuring sleep, and objective assessments, including actigraphy, pulse oximetry, sleep electroencephalography, and psychomotor vigilance test, provide more precise information on sleep disorders [12, 133, 135]. Although such studies are becoming more common, additional large cohort studies utilizing objective sleep measurements are warranted. This review suggests that introducing objective sleep measurements has been getting easier and more realistic in the real-world public health practice, as well as in the research context. Understanding which sleep-related indicators have been used in large population-based cohorts provides important context for interpreting sleep assessments in routine practice. This review supports clinicians and public health workers in making informed decisions when selecting and combining appropriate sleep-related indicators according to public health objectives.

One limitation of this study is that we only evaluated 19 large population-based cohorts in Japan. Notwithstanding, at least 20,000 “cohort” studies with any sample size (including hospital-based cohorts of patients with sleep disorders) have measured sleep parameters worldwide, and summarizing these data would be impractical. Therefore, restricting inclusion to cohorts with more than 10,000 participants and a follow-up period of at least 5 years was a pragmatic approach for the objectives of this review. In addition, we sought to compare some study results between cohort studies measuring sleep (e.g., sleep duration) subjectively and objectively. However, we could not find any two studies (with subjective or objective measures of sleep) setting the same outcome. Further examination of whether and how the sleep measurement methods could make a difference in the association between sleep (e.g., sleep duration) and health outcomes is warranted.

## Conclusions

Large population-based cohort studies in Japan have measured sleep subjectively, although the number of studies using objective measures is increasing. Future research should use objective sleep measures based on emerging technologies, such as portable electroencephalography, actigraphy, and smartphone apps, to improve the accuracy of sleep measurements and deepen our understanding of the effects of sleep on health.

## Abbreviations

3% ODI: 3% oxygen desaturation index
AIS: Athens Insomnia Scale
CIRCS: Circulatory Risk in Communities Study
ESS: Epworth Sleepiness Scale
ISI: Insomnia Severity Index
JACC: The Japan Collaborative Cohort
JECS: The Japan Environment and Children’s Study
J-MICC: Japan Multi-Institutional Collaborative Cohort
JNHS: Japan Nurses’ Health Study
J-EPISODE: The Japanese Epidemiological Study on Low-Dose Radiation Effects
JPHC: Japan Public Health Center
JPHC-NEXT: The JPHC for the Next Generation
MEQ: Morningness-Eveningness Questionnaire
PSQI: Pittsburgh Sleep Quality Index
TMM: The Tohoku Medical Megabank Project

## Supplementary information

**Additional file 1:** **Table S1.** List of individual reports on sleep in Japanese populations.

## Declarations

### Availability of data and material

There is no new data associated with this article.
