# Supplementary material for: A review of sleep-related indicators measured in large population-based cohort studies in Japan
Source: Environ Health Prev Med. 2026 Apr 3;31:28. doi: 10.1265/ehpm.25-00277 (PMC13057876; doi:10.1265/ehpm.25-00277)
Supplement: Supplementary file 1 — Additional file 1: Table S1. List of individual reports on sleep in Japanese populations. [file ehpm-31-028-s001.docx]

| Table S1. List of individual reports on sleep in Japanese populations. | | | | | | | | |
| --- | --- | --- | --- | --- | --- | --- | --- | --- |
| **Ref. no** | **Cohort** | **Authors** | **Year** | **Participants** | **Design** | **Outcome** | **Sleep-Related Data** | **Result** |
| 20 | JPHC | Weiderpass et al. | 2012 | 45,748 | Prospective | Epithelial ovarian cancer | Sleep duration (<6, 6–7, >7 hours per day, and as a continuous variable per hour) | Usual sleep duration of >7 hours per day was inversely associated with epithelial ovarian cancer risk. |
| 21 | JPHC | Svensson et al. | 2021 | 99,860 | Prospective | All-cause mortality and major cause-specific mortality | Sleep duration (≤5, 6, 7, 8, 9, and ≥10 hours per day) | Compared to the group with a sleep duration of 7 hours, an increased sleep duration was associated with a higher risk of mortality. |
| 22 | JPHC | Miyata et al. | 2024 | 36,854 | Prospective | Dementia requiring care | Sleep duration (3–5, 6, 7, 8, 9, and 10–12 hours per day) Changes in sleep duration from the baseline (≥2 hours decrease, 1 hour decrease, no change, an hour increase, and ≥2 hours increase) | A longer sleep duration and an increase of 2 or more hours in sleep duration over 5 years were associated with a higher risk of dementia compared to individuals with a sleep duration of 7 hours per day or those whose sleep duration remained unchanged. |
| 23 | JACC | Tamakoshi et al. | 2004 | 104,010 | Prospective | All-cause mortality | Sleep duration (≤4, 5, 6, 7, 8, 9, and ≥10 hours per day) | Sleep duration at night of 7 hours was found to show the lowest mortality risk. |
| 24 | JACC | Ikehara et al. | 2009 | 98,634 | Prospective | Mortality from cardiovascular disease and other causes | Sleep duration (≤4, 5, 6, 7, 8, 9, and ≥10 hours per day) | Both short and long sleep durations were associated with increased mortality from cardiovascular disease, noncardiovascular disease/noncancer, and all causes, forming a U-shaped relationship with the lowest risk at 7 hours. |
| 25 | JACC | Tamakoshi et al. | 2009 | 62,106 | Prospective | All-cause mortality | Sleep duration (6.5 to 7.4 hours per day or not) | Baseline healthy lifestyle combination was associated with a linear decrease in the risk of all-cause mortality. |
| 26 | JACC | Tamakoshi et al. | 2010 | 62,106 | Prospective | Life expectancy | Sleep duration (6.5 to 7.4 hours per day or not) | Among individuals aged 40 and 60 years, maintaining all 6 healthy lifestyle factors was associated with longer life expectancy. |
| 27 | JACC | Tanabe et al. | 2010 | 67,129 | Prospective | Mortality from cardiovascular disease, non-cardiovascular/non-cancer and external deaths | Napping or not Sleep duration (<6, 6, 7, 8, and ≥9 hours per day) | Daytime napping is associated with elevated risk of cardiovascular mortality as well as non-cardiovascular/non-cancer and external deaths. |
| 28 | JACC | Cao et al. | 2019 | 34,350 | Prospective | Breast cancer | Sleep duration (≤6, 7 and ≥8 hours per day) | Short sleep duration was associated with increased risk of incident breast cancer, especially among postmenopausal women and women with low parity. |
| 29 | JACC | Sakaniwa et al. | 2022 | 49,021 | Prospective | Life expectancy | Sleep duration (5.5 to 7.4 hours per day or not) | A sleep duration of 5.5 to 7.4 hours per day was expected to extend life expectancy by 1.3 to 1.7 years. |
| 30 | JACC | Arafa et al. | 2023 | 51,185 | Prospective | Liver cancer | Napping or not Sleep duration (<6, 6–8, and >8 hours per day) | Daytime napping was associated with a higher risk of liver cancer among older adults. |
| 31 | JACC | Okada et al. | 2023 | 20,318 | Prospective | Type 2 diabetes | Napping or not Sleep duration (≤5, 6, 7, 8, 9, and ≥10 hours per day) | Long sleep duration was associated with the risk of type 2 diabetes in both sexes, which was confined to the non-overweight. |
| 32 | JACC | Yan et al. | 2024 | 49,037 | Prospective | Gastric cancer | Napping or not Sleep duration (<6, 6–7, and ≥8 hours per day) | Daytime napping was associated with increased gastric cancer risk, especially among those who reported short sleep duration. |
| 33 | JACC | Guo et al. | 2025 | 95,174 | Prospective | Mortality from chronic kidney disease | Sleep duration (<6, 6–7, 7–8, 8–9, and ≥9 hours per day) | Sleep duration of 8 or more hours per day was associated with increased risk of chronic kidney disease mortality. |
| 34 | JACC | Shimizu et al. | 2024 | 67,269 | Prospective | Mortality from aortic disease | Napping or not Sleep duration (≤6, 7, and ≥8 hours per day) | Daytime napping but not sleep duration at night was associated with an increased risk of mortality from aortic disease. |
| 35 | Ohsaki | Kakizaki et al. | 2008 | 22,320 | Prospective | Prostate cancer | Sleep duration (≤6, 7-8, and ≥9 hours per day) | There was a significant inverse association between sleep duration and the risk of prostate cancer incidence. |
| 36 | Ohsaki | Kakizaki et al. | 2008 | 23,995 | Prospective | Breast cancer | Sleep duration (≤6, 7, 8, and ≥9 hours per day) | There was a significant inverse association between sleep duration and breast cancer risk, those who slept 6 hours or less having a significantly increased risk. |
| 37 | Ohsaki | Nagai et al. | 2013 | 13,629 | Cross-sectional | Weight gain (≥5kg) or body mass index ≥25kg/m^2^ | Sleep duration (≤5, 6–7, 8, and ≥9 hours per day) | Only obese long sleepers have a significantly increased long-term risk of ≥5kg weight gain. |
| 38 | Ohsaki | Kakizaki et al. | 2013 | 49,256 | Prospective | Cause-specific mortality | Sleep duration (≤6, 7, 8, 9, and ≥10 hours per day) | Except for stroke mortality, the association between long sleep duration and mortality was not modified by physical function or health status. |
| 39 | Miyagi and Ohsaki | Sugawara et al. | 2023 | 36,537 | Prospective | Endometrial cancer | Sleep duration (≤6, 7–8, and ≥9 hours per day) | There was no significant association between sleep duration and the incidence of endometrial cancer. |
| 40 | Takayama | Izuhara et al. | 2013 | 14,027 | Cross-sectional | Sleep disorders | PSQI | Sleep disorders were significantly associated with tinnitus. |
| 41 | Takayama | Kawachi et al. | 2016 | 27,896 | Prospective | Mortality from stroke | Sleep duration (≤6, 7, 8, and ≥9 hours per day) | Longer sleep duration was associated with increased mortality from total and ischemic stroke. Short sleep duration may be associated with a decreased risk of mortality from hemorrhagic stroke in men. |
| 42 | Takayama | Nagata et al. | 2023 | 3,090 | Prospective | Onset of menopause | Sleep duration (≤6, 7, 8, and ≥9 hours per day) | Short sleep duration is associated with later onset of menopause. |
| 43 | CIRCS | Tanigawa et al. | 2004 | 1,424 | Cross-sectional | Blood pressure | 3% ODI during sleep | There was a significant association between sleep-disordered breathing severity and blood pressure levels. |
| 44 | CIRCS | Tanigawa et al. | 2004 | 1,517 | Cross-sectional | Sleep-disordered breathing | 3% ODI during sleep | There was a significant positive association between usual alcohol consumption and sleep-disordered breathing. |
| 45 | CIRCS | Tanigawa et al. | 2006 | 1,763 | Cross-sectional | Atrial fibrillation | 3% ODI during sleep | There was a significant positive association between sleep-disordered breathing severity and the prevalence of atrial fibrillation. |
| 46 | CIRCS | Cui et al. | 2008 | 3,568 | Cross-sectional | Excessive daytime sleepiness and hypertension | 3% ODI during sleep Frequency of snoring, apnea, excessive daytime sleepiness, and morning sleepiness (three-point scale) | The severity of sleep-disordered breathing was significantly associated with excessive daytime sleepiness and hypertension. |
| 47 | CIRCS | Muraki et al. | 2010 | 4,398 | Prospective | Type 2 diabetes | 3% ODI during sleep | Nocturnal intermittent hypoxia was associated with increased risk of developing type 2 diabetes. |
| 48 | CIRCS | Muraki et al. | 2010 | 4,606 | Prospective | Metabolic syndrome | 3% ODI during sleep | Nocturnal intermittent hypoxia was associated with the accumulation of metabolic risk factors, especially among non-overweight individuals. |
| 49 | CIRCS | Muraki et al. | 2010 | 3,888 | Cross-sectional | C reactive protein | 3% ODI during sleep | Nocturnal intermittent hypoxia was associated with raised serum C reactive protein levels. |
| 50 | CIRCS | Yamagishi et al. | 2010 | 978 | Cross-sectional | Comparison of the sleep-disordered breathing prevalence | Respiratory disturbance index during sleep | The sleep-disordered breathing prevalence was lower among Japanese than the Americans, but the majority of the race/ethnic difference in the prevalence was explained by a difference in body mass index distribution. |
| 51 | CIRCS | Nagayoshi et al. | 2011 | 8,483 | Cross-sectional | Snoring | Frequency of snoring (four-point scale) | Body mass index, alcohol consumption, and cigarette smoking were positively associated with habitual snoring, especially in no overweight persons. |
| 52 | CIRCS | Cui et al. | 2011 | 3,113 | Cross-sectional | Sleep-disordered breathing | 3% ODI during sleep | Alcohol consumption was associated with the higher prevalence of sleep-disordered breathing. |
| 53 | CIRCS | Nagayoshi et al. | 2012 | 6,513 | Prospective | Cardiovascular disease | Frequency of snoring (four-point scale) | Self-reported habitual snoring was associated with increased risk of cardiovascular events. Overweight may partly mediate this association. |
| 54 | CIRCS | Onuki et al. | 2023 | 5,313 | Prospective | Cardiovascular disease | 3% ODI during sleep | Nocturnal intermittent hypoxia was associated with an increased risk of developing lacunar infarction and coronary heart disease. |
| 55 | J-MICC | Yamaguchi et al. | 2013 | 1,368 | Cross-sectional | Sleep-wake regularity | Sleep duration (<6, 6–7, and ≥7 hours per day) Sleep-wake regularity (good or poor) | Low protein intake, high carbohydrate intake, and irregular consumption of staple foods were significantly associated with poor sleep-wake regularity. |
| 56 | J-MICC | Kuriyama et al. | 2017 | 221 | Cross-sectional | Sympathetic nervous system activity and bone mass | Sleep duration (≥6 hours or not) PSQI (continuous) | Short sleep was associated with a decline in cortical bone thickness. |
| 57 | J-MICC | Katsuura-Kamano et al. | 2021 | 29,780 | Cross-sectional | Metabolic syndrome | Sleep duration (<6, 6–8, and ≥8 hours per day) | Skipping breakfast and short sleep duration were significantly associated with metabolic syndrome in men, and with obesity in both sexes. |
| 58 | J-MICC | Ogura et al. | 2022 | 3,880 | Cross-sectional | Metabolic syndrome | Regular bedtime and wake-up or not Sleep duration (<6, 6–7, and ≥7 hours per day) The average bedtime was categorized on <23:00, 23:00–24:00, and ≥24:00 | Irregular sleep was more strongly associated with metabolic syndrome than sleep duration or bedtime. |
| 59 | J-MICC | Omichi et al. | 2022 | 81,382 | Prospective | All-cause mortality | Regular bedtime and wake-up or not Sleep duration (<6, 6–8, and ≥8 hours per day) | Irregular sleep patterns were associated with a significantly increased risk of mortality across all categories, regardless of sleep duration. |
| 60 | J-MICC | Sogawa et al. | 2022 | 61,646 | Prospective | All-cause mortality | Regular hypnotics use or not | Hypnotic use was associated with a significantly greater risk of all-cause mortality, especially in males and participants aged <60 years. |
| 61 | J-MICC | Takagi et al. | 2023 | 2,233 | Cross-sectional | Falls | PSQI ≥6 or not | The combination of locomotive syndrome and a high PSQI score was an independent risk factor of falls. |
| 62 | J-MICC | Hama et al. | 2024 | 3,058 | Cross-sectional | Sleep quality | PSQI ≥6 or not Sleep duration (<6 hours per day or not)  Satisfaction (enough or not) Regular bedtime and wake-up or not | Dysphagia risk was associated with sleep quality in older individuals. |
| 63 | J-MICC | Kato et al. | 2024 | 2,246 | Cross-sectional | Locomotive syndrome | PSQI ≥6 or not | Poor sleep quality was independently associated with locomotive syndrome. |
| 64 | J-MICC | Morita et al. | 2024 | 2,044 | Cross-sectional | Daily sleep conditions | Sleep duration (minutes per day) Sleep efficiency (continuous) PSQI ≥6 or not ISI ≥10 or not MEQ (three groups) | Frequent forest walks were significantly associated with a lower prevalence of insomnia symptoms in women, but not in men. |
| 65 | JNHS | Chen et al. | 2015 | 27,370 | Cross-sectional | Lifestyle patterns | Sleep duration (≥6 hours per day or not) | Distinct lifestyle patterns were identified, and the pattern characterized by long working hours was significantly associated with shorter sleep duration and other unfavorable health behaviors. |
| 66 | JNHS | Hayashi et al. | 2022 | 15,019 | Cross-sectional | Poor memory or forgetfulness | Difficulty falling asleep or not Insomnia or not | Less sleep and night-shift work were significantly associated with the prevalence of severe complaints of reduced cognitive functioning. |
| 67 | JNHS | Terauchi et al. | 2024 | 9,728 | Cross-sectional | Excessive daytime sleepiness | ESS ≥11 or not | The effect of shift work on excessive daytime sleepiness differed by age, as shift work and obesity contributed to excessive daytime sleepiness only in older participants. |
| 68 | J-EPISODE | Kudo et al. | 2022 | 41,742 | Prospective | Mortality from cancer | Sleep quality (four-point scale) | Smoking, alcohol consumption, infrequent medical checkups, poor breakfast habits, inadequate sleep, and high or low body mass index were significantly associated with increased cancer mortality, whereas the risk from low-dose radiation, if any, was lower than these lifestyle factors. |
| 69 | J-EPISODE | Kudo et al. | 2022 | 43,692 | Prospective | Mortality from non-cancer | Sleep quality (four-point scale) | For many causes of non-cancer death, significantly high risks were observed for lifestyle factors such as smoking, alcohol consumption, frequency of medical examination, breakfast intake, sleep, and body mass index, but few for socioeconomic status and none for radiation. |
| 70 | JPHC -NEXT | Hanyuda et al. | 2021 | 106,282 | Prospective | Dry eye disease | Sleep duration (≤5, 6, 7, 8, 9, and ≥10 hours per day) Sleep quality (frequency of difficulty falling asleep, nocturnal awakenings, and morning fatigue) | Individuals with a shorter sleep duration or poor sleep quality had a higher prevalence of dry eye disease. |
| 71 | JPHC -NEXT | Hanyuda et al. | 2023 | 106,282 | Prospective | Dry eye disease | Sleep duration (≤5, 6, 7, 8, 9, and ≥10 hours per day) Sleep quality (frequency of difficulty falling asleep, nocturnal awakenings, and morning fatigue) | Individuals with a shorter sleep duration or poor sleep quality had a higher prevalence of dry eye disease. |
| 72 | JECS | Miyazaki et al. | 2016 | 3,873 | Cross-sectional | Allergic diseases | Children's sleeping place, sleeping posture, and regularity of bedtime | A significant association was observed between asthma and prone sleeping. |
| 73 | JECS | Morokuma et al. | 2017 | 8,631 | Prospective | Small for gestational age infants | Sleep duration (4–6, 6–7, 7–8, 8–9, 9–12 hours per day) Wake-up mood (five-point scale) | Neither the amount nor the quality of mothers' sleep was associated with the risk of small for gestational age birth. |
| 74 | JECS | Konishi et al. | 2019 | 103,099 | Cross-sectional | Changes in sleep-related variables | Wake-up time (continuous) Bedtime (continuous) Sleep duration (continuous) Quality of sleep (five-point scale) Depth of sleep (five-point scale) | Maternal sleep state differed by age, with younger women experiencing more sleep trouble and feeling less rested compared to other groups. |
| 75 | JECS | Sugimori et al. | 2019 | 72,624 | Prospective | Sleep durations in infants | Infant sleeping duration (<11 hours per day or not) | Maternal intake of fermented food, especially miso, during the pregnancy was independently associated with reduced risk of infant short sleep. |
| 76 | JECS | Myoga et al. | 2019 | 48,787 | Prospective | Gestational diabetes | Sleep duration (<5, 5–7, 7–10, and ≥10 hours per day) | Abnormally long or short sleep duration was associated with higher random blood glucose levels and an increased risk of positive gestational diabetes screening in pregnant women. |
| 77 | JECS | Nakahara et al. | 2020 | 81,821 | Prospective | Preterm birth, infant sleep, and temperament | Sleep duration (<6, 6–7, 7–8, 8–9, 9–10, and >10 hours per day) Bedtime (21:00 to midnight, midnight to 3:00, and others) Depth of sleep (five-point scale) Feeling when waking up in the morning (three-point scale) Five or more awakenings during the night in infant Infant sleeping longer during the day than at night or not | Maternal sleep problems before and during pregnancy were associated with preterm birth and child sleep problems and temperament. |
| 78 | JECS | Nakahara et al. | 2020 | 62,612 | Prospective | Sleeping problems in infants | Infant waking up ≥3 times in a night or not Infant waking up ≥1 times and remaining awake for more than 1 hour or not  Infant sleep duration (<8 hour during the night or not) Infant sleep at ≥22:00 or not Infant crying for ≥5 days in a week or not | False-positive non-reassuring fetal status was associated with sleep problems in 1-year-old infants, including shorter nighttime sleep duration, frequent night-time crying, and late bedtime. |
| 79 | JECS | Murata et al. | 2021 | 82,171 | Prospective | Low birth weight infants, small for gestational age infants, and macrosomia | Maternal sleep duration (<6, 6–7, 7–8, 8–9, 9–10, and 10-12 hours per day) | Maternal sleep duration of 9.0–9.9 hours was significantly associated with the decreased incidence of low-birth-weight infants and small for gestational age infants in pregnant women with appropriate gestational weight gain, compared with that of 6.0–7.9 hours. |
| 80 | JECS | Nakahara et al. | 2021 | 73,827 | Prospective | Sleeping and developmental problems in infants | Sleep duration (<6, 6–7, 7–8, 8–9, 9–10, and >10 hours per day) Bedtime (21:00 to midnight, midnight to 3:00, and others) Depth of sleep (five-point scale) Feeling when waking up in the morning (three-point scale) Infant waking up ≥5 times in a night in infants or not Infant sleeping longer during the day than at night or not | Maternal short sleep and late bedtime before and during pregnancy increased occurrence of offspring's sleeping disturbances. |
| 81 | JECS | Nakahara et al. | 2021 | 76,368 | Prospective | Sleeping and developmental problems in infants | Sleep duration (<6, 6–7, 7–8, 8–9, 9–10, and >10 hours per day) Bedtime (9:00 to midnight, midnight to 3:00, and others) Depth of sleep (five-point scale) Feeling when waking up in the morning (three-point scale) Infant waking up ≥5 times in a night in infants or not Infant sleeping longer during the day than at night or not | Higher levels of maternal physical activity, both before and during pregnancy, were associated with a reduced risk of sleep and developmental problems in 1-year-old infants. |
| 82 | JECS | Sugimori et al. | 2022 | 87,337 | Prospective | Sleep durations in infants | Infant sleep duration (<11 hours per day or not) | Low fish intake during pregnancy was associated with an increased risk of infants sleeping less than 11 hours at 1 year of age. |
| 83 | JECS | Nakahara et al. | 2022 | 66,935 | Prospective | Bedtime in infants | Infant waking up ≥3 times in a night in infants or not Infant waking up ≥1 times and remaining awake for more than 1 hour in infants or not  Infant sleep duration (<8 hour during the night or not) Infant sleep at ≥22:00 or not Infant crying for ≥5 days in a week or not | Both low and high levels of maternal hemoglobin during pregnancy were associated with a higher risk of late bedtime in 1-year-old infants. Additionally, low maternal hemoglobin levels were associated with abnormal fine motor development. |
| 84 | JECS | Nishioka et al. | 2022 | 74,525 | Prospective | Sleep duration and bedtime in children | Infant sleep duration (<11 hours per day or not) (1-year-olds) Infant sleep duration (<10 hours per day or not) (3-year-olds) | Use of portable electronic devices, but not television or digital versatile disc viewing, was associated with shorter sleep duration in infants. The risk increased with longer use of portable devices. Late bedtime at age 1 year was associated with late bedtime at age 3 years. |
| 85 | JECS | Nakahara et al. | 2022 | 69,969 | Prospective | Autism spectrum disorder in infants | Sleep duration (<6, 6–7, 7–8, 8–9, 9–10, and >10 hours per day) Bedtime (9:00 to midnight, midnight to 3:00, and others) | Maternal physical activity and sleep duration during pregnancy were associated with the risk of autism spectrum disorder in 3-year-old infants. |
| 86 | JECS | Inoue et al. | 2022 | 64,200 | Prospective | Sleep durations in children | Infant sleep duration (<10 hours per day or not) (3-year-olds) | The consumption of fermented foods during pregnancy was associated with reduced risk of sleep deprivation in 3-year-old children. |
| 87 | JECS | Kikuchi et al. | 2022 | 80,970 | Cross-sectional | Temperament in infants | Infant waking up ≥5 times in a night in infants or not Infant sleeping longer during the day than at night or not | Sleep quality was specifically associated with temperament in one-month-old infants. |
| 88 | JECS | Inoue et al. | 2022 | 65,210 | Prospective | Sleep durations in children | Infant sleep duration (<11 hours per day or not) (1-year-olds) Infant sleep duration (<10 hours per day or not) (3-year-olds) | Although the frequency of yogurt and cheese intake at age 1 was not associated with sleep duration at age 1 or 3, a trend test showed a significant association between yogurt intake frequency at age 1 and sleep duration at age 3. |
| 89 | JECS | Tsuchiya et al. | 2022 | 90,148 | Prospective | Bruxism in children | Infant sleep duration (7 one-hour categories by age in months) | The sleep duration in infant stage, especially during newborn stage was associated with the prevalence of bruxism behavior in children. |
| 90 | JECS | Kikuchi et al. | 2023 | 69,751 | Prospective | Autism spectrum disorder in infants | Infant waking up ≥5 times in a night in infants or not Infant sleeping longer during the day than at night or not | Infants with longer daytime sleep had a higher risk of later autism spectrum disorder than those with shorter daytime sleep. |
| 91 | JECS | Hotta et al. | 2023 | 77,876 | Prospective | Sleep-and-wakefulness states in infants | Sleep duration (continuous) at 1 month, 1.5, and 3 years of age Sleep onset and sleep offset (continuous) at 1.5 and 3 years of age | Longer phototherapy duration was associated with reduced total sleep time at 1 month of age, indicating a temporary impact on infant sleep duration. |
| 92 | JECS | Sato et al. | 2024 | 91,497 | Prospective | Sleep durations in children | Infant seep durations (continuous) | No association was found between isolated orofacial clefts and sleep duration at 1 month, 6 months, 1 year, or 3 years of age. Children with isolated orofacial clefts had sufficient average sleep duration at each age. |
| 93 | JECS | Kikuchi et al. | 2024 | 63,418 | Prospective | Autism spectrum disorder in children | Infant sleeping longer during the day than at night or not (1-month-olds) Infant sleep duration (<8 hours per day or not) (6-months and 1-year-olds) Infant waking up ≥5 times in a night in infants or not (1-month-olds) Infant waking up ≥4 times in a night in infants or not (6-month-olds) Infant waking up ≥3 times in a night in infants or not (1-year-olds) | Later short sleep onset and earlier recovery were associated with lower autism spectrum disorder risk. |
| 94 | JECS | Kawai et al. | 2024 | 52,140 | Prospective | Height in children | Infant nighttime sleep duration (≤9, 9–10, 10-11, and ≥11.5 hours per day) Infant total sleep duration (≤11, 11.5, 12, 12.5, 13, and ≥13.5 hours per day) | Longer nighttime sleep duration at 1.5 years of age was associated with taller stature at 3 years, whereas total sleep duration was not. |
| 95 | JECS | Kikuchi et al. | 2024 | 85,731 | Cross-sectional | Otitis media | Infant sleep position (supine, lateral, prone, or not determined) | Infants aged 6 months or younger who slept in the prone position were more likely to have otitis media. |
| 96 | Nagahama | Yoshimura et al. | 2012 | 5,980 | Cross-sectional | Nocturnal voiding | Seep durations (continuous) Sleeplessness or not Daytime sleepiness or not | Plasma BNP was associated with the prevalence and severity of nocturnal voiding, alongside known factors such as age, urgency, sleep quality and quantity, and obesity. |
| 97 | Nagahama | Murase et al. | 2015 | 9,804 | Cross-sectional | Sleep duration and quality | Sleep duration (<5, 5–6, 6–7, 7–8, and >8 hours per day) Satisfaction (poor or not) Regular bedtime and wake-up or not | Knee and low back pains were independently associated with short sleep duration and poor sleep quality. |
| 98 | Nagahama | Matsumoto et al. | 2017 | 9,788 | Cross-sectional | Non-restorative sleep | Non-restorative sleep (yes or no) Regular bedtime and wake-up or not Sleep duration (<5, 5–6, 6–7, 7–8, and >8 hours per day) Daytime sleepiness (three-point scale) Regular hypnotics use or not | Sleep-related factors were independently associated with subjective non-restorative sleep. A composite score incorporating these and other factors was linearly associated with the frequency of non-restorative sleep complaints. |
| 99 | Nagahama | Matsumoto et al. | 2018 | 7,051 | Cross-sectional | Diabetes and hypertension | Sleep duration (continuous) ESS 3% ODI during sleep | Sleep-disordered breathing and obesity, but not short sleep duration, were independently associated with diabetes and hypertension, with differences in risk observed by gender and menopausal status. |
| 100 | Nagahama | Fukunaga et al. | 2019 | 5,297 | Prospective | Lower urinary tract symptoms | Sleep quality (poor or not) Sleep restriction (<5 or ≥5 hours per night) Hypnotics use or not | Sleep disturbance was significantly associated with the presence of lower urinary tract symptoms in a cross-sectional manner and with worsening lower urinary tract symptoms longitudinally. |
| 101 | Nagahama | Matsumoto et al. | 2019 | 5,854 | Cross-sectional | Nocturnal blood pressure | Sleep duration (continuous) Sleep efficiency (continuous) Fragmentation index (continuous) | Low sleep efficiency was a strong determinant for increased sleep blood pressure and decreased nocturnal blood pressure drop. |
| 102 | Nagahama | Tabara et al. | 2019 | 5,683 | Cross-sectional | Nocturnal urination frequency | Sleep duration (continuous) PSQI (usual bedtime) Physician-diagnosed sleep apnea syndrome | Coffee and green vegetable consumption and later bedtime but not sleep duration were lifestyle factors associated with nocturnal urination frequency. |
| 103 | Nagahama | Matsumoto et al. | 2020 | 7,713 | Cross-sectional | Sleep disordered breathing | Sleep duration (continuous) 3% ODI during sleep ESS (>10 or not) PSQI (≥6 or not) | Metabolic comorbidities contributed to sleep-disordered breathing regardless of the degree of obesity. |
| 104 | Nagahama | Hamada et al. | 2021 | 7,151 | Cross-sectional | Sleep disordered breathing | Sleep duration (continuous) 3% ODI during sleep ESS (≥11 or not) Frequency of snoring and stop breathing (three-point scale) | Night-time frequency of urination was independently associated with sleep-disordered breathing. |
| 105 | Nagahama | Minami et al. | 2021 | 5,736 | Cross-sectional | Diabetes | Sleep duration (<5, 5–7, and ≥7) 3% ODI during sleep ESS (continuous) | Moderate-to-severe sleep disordered breathing was associated with diabetes risk in females with a family history of diabetes. |
| 106 | Nagahama | Nakatsuka et al. | 2021 | 7,729 | Cross-sectional | Cardiovascular disease | Sleep duration (continuous) 3% ODI during sleep | Sleep disordered breathing severity was associated with the maximum common carotid artery intima-media thickness level. |
| 107 | Nagahama | Takahashi et al. | 2022 | 6,908 | Cross-sectional | Sleep duration | Sleep duration (continuous) PSQI (subjective sleep duration) ESS (≥11 or not) Nonrestorative sleep (yes or no) 3% ODI during sleep | Subjective sleep duration exceeded objective duration when actual sleep was under 7 hours but was shorter when sleep exceeded 7 hours. In sleep-disordered breathing, subjective duration was longer than objective duration, increasing with severity. |
| 108 | Nagahama | Murase et al. | 2022 | 6,568 | Cross-sectional | Urinary albumin excretion | Sleep duration (continuous) 3% ODI during sleep Sleep efficiency (continuous) | Sleep-disordered breathing was independently associated with increased urinary albumin excretion beyond the effects of blood pressure. |
| 109 | Nagahama | Matsumoto et al. | 2023 | 7,680 | Cross-sectional | Hemoglobin A1c levels | Sleep duration (continuous) 3% ODI during sleep ESS (≥11 or not) PSQI (≥6 or not) | Higher hemoglobin A1c levels were independently associated with moderate-to-severe obstructive sleep apnea in individuals without antidiabetic treatment. |
| 110 | Nagahama | Negoro et al. | 2023 | 8,076 | Cross-sectional | Nocturia | Sleep quality (poor or not) Hypnotics use or not | Nocturia and poor sleep associated with each other. |
| 111 | Nagahama | Tabara et al. | 2023 | 3,844 | Cross-sectional | Masked hypertension | Sleep duration (continuous) 3% ODI during sleep Sleep efficiency (continuous) | Sleep-related factors associated with masked hypertension differed depending on the presence of sleep hypertension. |
| 112 | Nagahama | Nakatsuka et al. | 2023 | 6,373 | Cross-sectional | Metabolome | Sleep duration (continuous) 3% ODI during sleep | Moderate-to-severe sleep-disordered breathing was independently associated with higher plasma fructose levels after adjustment for confounding factors. |
| 113 | Nagahama | Sunadome et al. | 2023 | 7,895 | Cross-sectional | Serum uric acid | Sleep duration (continuous) 3% ODI during sleep | Serum uric acid levels were positively associated with the severity of sleep-disordered breathing. |
| 114 | Nagahama | Nishi et al. | 2024 | 7,948 | Prospective | Asthma | Sleep duration (continuous) 3% ODI during sleep History of sleep apnea syndrome | Elderly patients with new-onset asthma had more frequent comorbidity of moderate to severe sleep disordered breathing than those non-elderly. |
| 115 | TMM Cohort study | Hirata et al. | 2020 | 904 | Cross-sectional | Hypertension | Sleep efficiency (continuous) | Reduced sleep efficiency was significantly related to an increased prevalence of hypertension. |
| 116 | TMM Cohort study | Hirata et al. | 2021 | 1,384 | Cross-sectional | Hypertension | Sleep efficiency (continuous) | The impact of the overall risk factors, including the urinary Na/K ratio and sleep efficiency, on home hypertension was higher than that of conventional risk factors alone. |
| 117 | TMM Cohort study | Miyagawa et al. | 2022 | 14,329 | Cross-sectional | Daytime sleepiness | Sleep duration (<5, 5–6, 6–7, 7–8, 8–9 and ≥9) Frequency of daytime sleepiness (four-point scale) | Genetic variants in the orexin receptor 2 gene were associated with self-reported daytime sleepiness. |
| 118 | TMM Cohort study | Matsuyama et al. | 2022 | 8,440 | Cross-sectional | Depression | AIS (continuous) | Cutoffs based on estimated prevalence from background factors and insomnia scores reduced the number of people screened without decreasing the number of detected depression cases. |
| 119 | TMM Cohort study | Murakami et al. | 2022 | 17,586 | Cross-sectional | Insomnia | AIS (≥6 or not) | Social isolation from family and friends was associated with increased risks of insomnia. |
| 120 | TMM Cohort study | Li et al. | 2023 | 154 | Cross-sectional | Sleep–wake conditions | Sleep–wake conditions (three-point scale) | Machine learning models using heart rate variability predicted sleep–wake conditions during pregnancy with high accuracy. |
| 121 | TMM Cohort study | Akaishi et al. | 2024 | 62,796 | Cross-sectional | Depressive state and sleep disturbance | AIS (≥6 or not) | Low hemoglobin levels and elevated inflammatory hematological ratios were associated with depressive state and sleep disturbances. |
| ^*^ Summarized in Japanese from Reference No. 75. Abbreviation: JPHC, Japan Public Health Center-based prospective Study; JACC, The Japan Collaborative Cohort Study; CIRCS, Circulatory Risk in Communities Study; J-MICC, Japan Multi-Institutional Collaborative Cohort Study; JNHS, Japan Nurses' Health Study; JEPISODE, Japanese epidemiological study on low-dose radiation effects; JECS, Japan Environment and Children's Health; TMM, Tohoku Medical Megabank; PSQI, Pittsburgh sleep quality index; ODI, oxygen desaturation index; ISI, insomnia severity index; MEQ, morningness–eveningness questionnaire; ESS, Epworth sleepiness scale; BNP, B-type natriuretic peptide; AIS, Athens Insomnia Scale. | | | | | | | | |
